# Supplementary material for: Robot-Assisted Approach to Diabetes Care Consultations: Enhancing Patient Engagement and Identifying Therapeutic Issues
Source: Medicina (Kaunas). 2025 Feb 17;61(2):352. doi: 10.3390/medicina61020352 (PMC11857797; doi:10.3390/medicina61020352)
Supplement: Supplementary file 1 [file medicina-61-00352-s001.zip › medicina-3459039-supplementary.pdf]

medicina MDPI

Supple Table 1 Response phrases by the RoBoHoN during interviews and corresponding Japanese words

I see.<br>なるほど	Right.<br>そうだよねー
That's good.<br>それがいいよね	Yes, indeed.<br>そんなことってあるね
Is that so.<br>そうなんですね	It really is.<br>なかなかねー
I see it's been on your mind as well.<br>自分でも気になっているんだよね	Was this question difficult to answer?<br>この質問は、答えるのが難しかったかな？
Really?<br>そうなの？	It sounds as though you don't see that as a good thing…<br>それは、良いことだとは思っていないように聞こえるけど…
Please tell me about your current situation, in a little more detail.<br>今のことを、もう少し詳しく聞かせて	Oh wow, I never would have thought.<br>へーっ そんなことがあったんだね
Did you ever wish you had more support?<br>もっと協力してほしいと思ったことは、ないのかな？	Could you tell me why?<br>理由があれば、教えて欲しい
Indeed, it's not just about you.<br>自分ひとりというわけではないからねー	How do you cope in those situations?<br>そんな時はどうしているか教えてね
Please tell me the specific amount, and other details.<br>具体的な量などを教えてね	Thank you for sharing this information with me.<br>お話し聞かせてくれて、ありがとう
That's wonderful!<br>すてきだと思ったー！	You've certainly been doing your best.<br>これまで頑張っていたんだね
I will discuss this with your GP.<br>主治医の先生に相談するね	I will ask your GP, in an indirect way.<br>先生にそれとなく聞いて

GP, general practitioner
